# Elderly Men Have Low Levels of Anti-Müllerian Hormone and Inhibin B, but with High Interpersonal Variation: A Cross-Sectional Study of the Sertoli Cell Hormones in 615 Community-Dwelling Men

**DOI:** 10.1371/journal.pone.0070967

**Published:** 2013-08-05

**Authors:** Yih Harng Chong, Nicola A. Dennis, Martin J. Connolly, Ruth Teh, Gregory T. Jones, Andre M. van Rij, Stephanie Farrand, A. John Campbell, Ian S. MLennan

**Affiliations:** 1 Department of Anatomy, Otago School of Medical Sciences, University of Otago, Dunedin, New Zealand; 2 Department of Medicine, Dunedin School of Medicine, University of Otago, Dunedin, New Zealand; 3 Department of Surgical Sciences, Dunedin School of Medicine; University of Otago, Dunedin, New Zealand; 4 Brain Health Research Centre, University of Otago, Dunedin, New Zealand; 5 Freemason’s Department of Geriatric Medicine, University of Auckland, Auckland, New Zealand; 6 Department of General Practice and Primary Health Care, University of Auckland, Auckland, New Zealand; Azienda Policlinico S. Orsola-Malpighi, Italy

## Abstract

The Sertoli cells of the testes secrete anti-Müllerian hormone (Müllerian inhibiting Substance, AMH) and inhibin B (InhB). AMH triggers the degeneration of the uterine precursor in male embryos, whereas InhB is part of the gonadal-pituitary axis for the regulation of sperm production in adults. However, both hormones are also putative regulators of homeostasis, and age-related changes in these hormones may therefore be important to the health status of elderly men. The levels of AMH in elderly men are unknown, with limited information being available about age-related changes in InhB. We have therefore used ELISAs to measure Sertoli cell hormone levels in 3 cohorts of community-dwelling men in New Zealand. In total, 615 men were examined, 493 of which were aged 65 or older. Serum AMH and InhB levels inversely correlated with age in men older than 50 years (p<0.001) but not in the younger men. A minority of elderly men had undetectable levels of AMH and InhB. The variation in hormone levels between similarly aged men increased with the age of men. AMH and InhB partially correlated with each other as expected (r = 0.48, p<0.001). However, the ratio of the two Sertoli hormones varied significantly between men, with this variation increasing with age. Elderly men selected for the absence of cardiovascular disease had AMH levels similar to those of young men whereas their InhB levels did not differ from aged-matched controls. These data suggests that Sertoli cell number and function changes with age, but with the extent and nature of the changes varying between men.

## Introduction

The testes contain two distinct endocrine cells, the androgenic Leydig cells and the Sertoli cells, which produce AMH and InhB. In male embryos, AMH triggers the degeneration of the uterine precursor [1], but the testes continue to secrete AMH throughout development and into adulthood [2], [3]. The AMH in blood has been presumed to be non-functional. However, its pattern of secretion is regulated, with conservation between species, ranging from fish to man [4]. This suggests that AMH may have undetected functions in animals and humans, as non-functional gene output degrades across evolutionary time. Consistent with this, AMH has been implicated in generating the male bias in parts of the murine nervous system [5], [6], [7] and lungs [8].

Recent studies also implicate serum AMH in human non-gonadal development. The level of AMH varies greatly between boys of similar age, with the interpersonal differences remaining stable over years [3], [9]. An individual boy’s level of AMH inversely correlates with his rate of maturation [9]. Similarly, in boys with an autistic spectrum disorder, their levels of AMH correlate with the breadth of their symptoms [10]. Men have lower levels of circulating AMH than boys [3], but the levels of AMH in men are nevertheless sufficient to effect responsive cells in vitro [11]. This suggests that AMH may have functions in adults, with regulation of the cardiovascular system being one emerging possibility [12], [13].

InhB is part of the gonadal pituitary axis and contributes to the regulation of sperm production [14]. Like testosterone, the actions of InhB appear to extend beyond primary reproduction to homeostasis. For example, the inhibins regulate bone, with the onset of osteoporosis in women being linked to declining levels of Inhibins during the menopausal transition [15]. Boys also produce InhB, despite their gonadal-pituitary being largely quiescent during childhood and their testes lacking sperm [16]. The function(s) of InhB in boys is unknown, but potentially important, as the levels of InhB in autistic boys are strongly and positively correlated with the breadth of their symptoms [10].

The classical functions of both AMH and InhB are independent of each other. However, AMH and InhB cooperate to suppress testicular cancer in mice [17]. In other circumstances, their effects on downstream signaling will generally be antagonistic [18]. Thus, for some functions the relative levels of AMH and InhB may have biological significance.

As noted above, both Sertoli cell hormones have well characterized classical functions, but with little exploration of their wider functions. Consequently, much of the basic information relating to these hormones is absent, including their circulating profiles across the lifespan of men. Ovarian production of both AMH and InhB decline during the menopausal transition, and is absent after the menopause. Testosterone secretion declines with age in most men [19], but with an average effect that is much less profound than the loss of ovarian steroids. Sertoli cell number declines with age [20], [21], but the effect of this on AMH production in elderly men is unknown, with only minimal information being available about InhB levels in elderly men [22].

This paucity of basic information makes it difficult to determine whether changes in Sertoli cell hormones might contribute to the deterioration of elderly men, and/or whether sex differences in age-related diseases are partially due the loss of gonadal protein hormones. We have therefore examined the levels of AMH and InhB, and the ratio of these two hormones, to produce the first description of Sertoli cell function in elderly men. We report that, on average, both Sertoli cell hormones are lower in elderly men, with wide variation between the two hormones suggesting age-related changes in both the regulation and the number of Sertoli cells.

## Materials and Methods

### Ethics

All study participants gave written informed consent. The study of the Otago elderly subset cohort, and Vascular cohort were both approved by the Lower South Regional Ethics Committee of New Zealand, whilst the LiLACS NZ cohort study was approved by the Northern X Regional Ethics Committee of New Zealand. The younger Otago men study was approved by the University of Otago Human Ethics Committee.

### Study Populations

Serum samples were collected from 615 men aged from 19 to 93 years, with a focus on elderly men (n = 496). The participants were drawn from three distinct cohorts (Table 1). A non-fasting 5 mL venous blood sample was obtained in the antecubital vein between 7 am and 3 pm. The blood was clotted, and the serum aliquoted and stored at −80 C.

**Table 1:** Characteristics of participants in three cohorts.

	Otago	Vascular	LILACS
n	205	153	257
**Mean age (age range) years**	59 (19–90)	71.7 (54–93)	84.3 (80–90)
**AMH Mean (sd) pM**	23.0 (16.9)	26.7 (16.6)	14.4 (14.0)
**AMH median pM**	19.1	22.7	12.1
**AMH 2.5^th^ –97.5^th^ centile pM**	0.9–73.4	3–73.2	0–52.9
**Proportion less than 2.5^th^ centile young men’s AMH**		11 (7.2%)	92 (35.8%)
**InhB Mean (sd) pg/mL**	137.6 (68.6)	94.2 (61.4)	108.7 (80.2)
**InhB median pg/mL**	131.2	80.6	100.9
**InhB 2.5^th^ –97.5^th^ centile pg/mL**	7.8–276.8	11.5–251.3	2.4–321.2
**Proportion less than 2.5^th^ centile young men’s InhB**		44 (29%)	66 (26%)

For AMH, to convert pmol/L to ng/mL, divide by 7.14.

The Otago cohort was a cross-sectional study of 99 community dwelling men aged 70 or older, with 119 younger men as a comparison. The men were predominantly Caucasian and resided in Dunedin, a small university city in New Zealand. The exclusion criteria for this cohort was limited to a history of testicular tumor, testicular trauma, orchidectomy, undescended testes, self-reported infertility, chemotherapy or abdominal or pelvic radiation therapy. None of the men were on anti-androgen medication or steroid medications. The elderly men were recruited from community groups and clubs, including men receiving outpatient rehabilitation for physical or mental health (mood disorders or cognitive decline). The younger men were recruited from various facilities including staff and students at the local university, medical and non-medical hospital staff, light and heavy industrial occupation, civil service, and office-based private sector employees.

The vascular cohort was a distinct Dunedin cohort comprised of 153 community dwelling men aged 54–93 years, with no history of cardiovascular disease. These men were recruited from advertisements and screened for peripheral arterial disease and carotid stenosis. Men with a carotid stenosis of greater than 50% and men with an ankle-brachial pressure index of less than 0.9 or greater than 1.3 were excluded from this cohort. Their sera were obtained from the Vascular Research Group Biobank, University of Otago.

Sera from 257 elderly men were analyzed from the LiLACS NZ Research Group (Life and Living in Advanced Age: A Cohort Study in New Zealand; Te Puawaitanga o Nga Tapuwae Kia Ora Tonu). This is an ongoing epidemiological study investigating factors that lead to successful aging in elderly New Zealanders [23]. A cohort of 937 community-dwelling men and women were recruited in the East Coast (Bay of Plenty and Rotorua) of the North Island, from the electoral roll registry, with methodology described elsewhere [24]. Inclusion criteria were primarily based on age and ethnicity: M*ā*ori aged between 80–90 years old, and non-M*ā*ori aged 85 years old were recruited, with a response rate from men of 65%. Study participants had varying health and physical performance status measured, with the 257 men examined being those that had consented for their sera to be used for collaborative research. None of the participants reported a history of orchidectomy.

### Laboratory Analysis

Serum AMH levels were measured in duplicate, with 575 of the 615 samples being measured with the enzyme immunoassay AMH/MIS kit (Beckman Coulter, Fullerton, CA; A16507, analytical sensitivity 1 pmol/L, 0.14 ng/ml). The other 40 were analyzed MIS/AMH ELISA (Diagnostic System Laboratories, Webster, TX; DSL-10-14400, analytical sensitivity 0.04 pmol/L, 0.006 ng/ml), which became unavailable after Beckman Coulter acquired DSL. The two kits give similar results [25], which was confirmed in our laboratory by analyzing over 100 samples unrelated to the current study, with both ELISA kits. The intra and interassay coefficient of variation (CV) were 8.8% and 10.3%.

Serum InhB levels was measured in duplicate by immunoassay (Beckman Coulter; A81303, with analytical sensitivity of 2.6pg/mL). The intra and interassay coefficient of variation (CV) were 6.5% and 12.6%.

### Calculations

The levels of the two Sertoli cell hormones varied between individuals. If this variation is examined using a simple ratio of the hormones (AMH/InhB) then the resulting distribution will be non-symmetrical. Individuals with high AMH will be represented on a scale from 1 to infinity whereas those with high InhB will have values between 0 and 1. The ratio was therefore calculated using the ratio of the natural logs of the hormones (ln(AMH)-ln(InhB)) which produces a symmetrical distribution, without bias to either hormone. When this ratio is standardized using the mean levels of the hormones in the young men, then the distribution centers around zero. Any variation from a normal distribution indicates biological skewing, with the width of the bell curve being a measure of the variation in the population: as per the normal distribution of each individual hormone.

### Statistical Analysis

The associations between AMH, InhB and age were tested by linear regression. The AMH and InhB levels in the Otago cohort were compared with the data from the other two cohorts using the nonparametric Wilcoxon-Mann-Whitney test. Skewness was calculated and its significance tested using the SKTEST. All calculations were performed in Stata/IC 12.1 (StataCorp LP, College Station, TX).

## Results

The levels of Sertoli cell hormones in a cross-section of community dwelling men (n = 205, Otago cohort) were examined, with the sole exclusion criteria being pharmacological or physical manipulation of the testes.

### AMH in Community-dwelling Men

**Figure 1:**
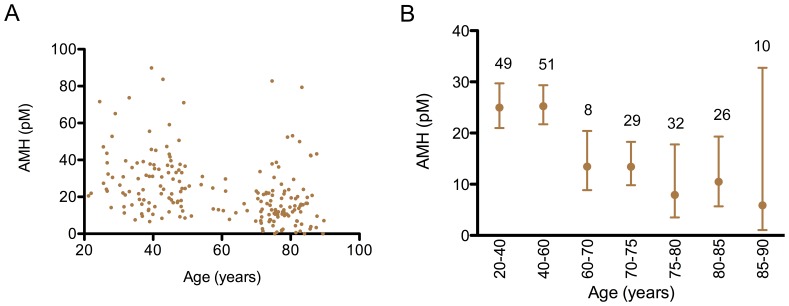
A cross-sectional study of the AMH levels across the adult lifespan of males. The levels of serum AMH in 205 community dwelling men (Otago cohort) are illustrated. A: each dot represented an individual. B: the geometric mean and 95% confidence interval is indicated for various age groups. The levels of AMH in the older men (80–93 years) were significantly less than those in the younger men (20–40 years) (p = 0.006). To convert pmol/L of AMH to ng/ml, divide by 7.14.

The levels of AMH were highly variable between men, with a distribution that was positively skewed in both the younger and older men (Fig. S1). All younger men had AMH levels that were greater than 6.5 pmol/L (0.91 ng/ml). There was no effect of age on the levels of AMH in younger men (r = −0.05, n = 95, <50 years), after which there was a change in the profile of AMH levels (Fig. 1A). In the older men, there was a significant and progressive decline in the geometric mean for AMH, with a simultaneous increase in variability between the men: AMH was undetectable in 5% of the elderly men, with 10% having high levels of AMH, above the 95% confidence interval of the younger men (Fig. 1B). Consequently, the coefficient of variation (%CV) increased with age.

### Inhibin B in Community-dwelling Men

**Figure 2:**
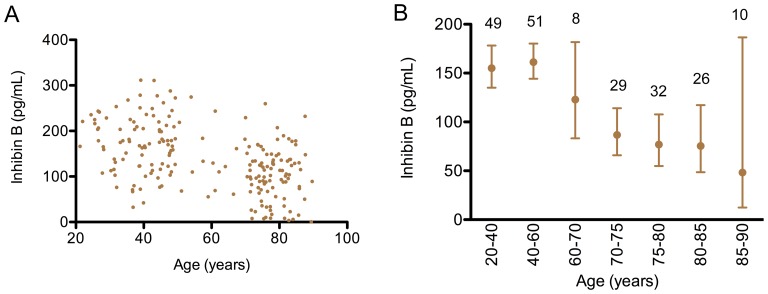
A cross-sectional study of the serum InhB levels across the adult lifespan of males. The levels of serum InhB in 205 community dwelling men (Otago cohort) are illustrated. A: each dot represented an individual. B: the geometric mean and 95% confidence interval is indicated for various age groups. The levels of Inhibin B in the older men (80–93 years) were significantly less than those in the younger men (20–40 years) (p<0.001).

The profile of InhB was similar to that of AMH. InhB was less variable between individuals than AMH (cf Fig. 1A, 2A), and was normally distributed in the younger men (Fig. S2). An age-related change in InhB was only observed in the elderly. As with AMH, this involved a diminishing geometric mean with age, with a concurrent increase in CV (Fig. 2B). 20% of the elderly men had levels of InhB below 51 pg/ml (2.5th centile of the younger men).

### Hormone Balance in Community-dwelling Men

**Figure 3:**
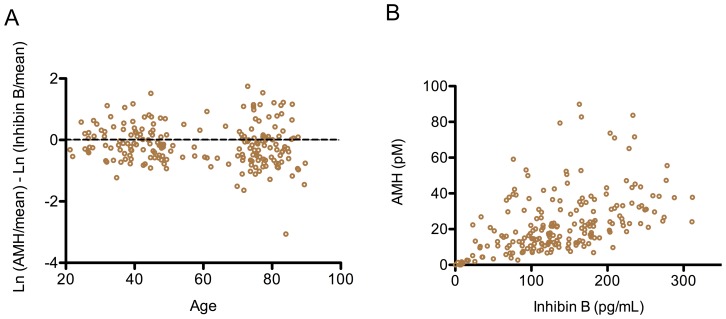
A cross-sectional study of the balance of Sertoli cell hormones across the lifespan of men. The relationship between AMH and InhB in 205 community dwelling men (Otago cohort) is illustrated. A: The ratio of the hormones across the age spectrum was calculated as described in the Methods. B: Each open circle represents an individual. The linear regression between AMH and Inhibin B was r = 0.48 (p<0.001).

The relative levels of AMH and InhB varied between individuals, in both the young and the elderly (Fig. 3A). However, the levels of the two hormones were not completely independent as a linear regression between them was r = 0.48, p<0.001 (Fig. 3B).

**Figure 4:**
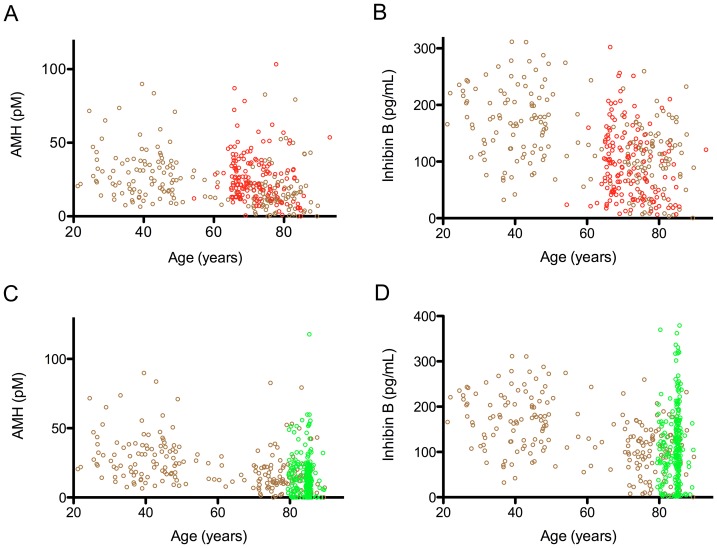
A comparison of Sertoli hormone levels between 3 New Zealand cohorts. The Otago cohort (205 community dwelling men in Dunedin) is illustrated with brown open circles; the Vascular cohort (153 men in Dunedin selected for their absence of cardiovascular disease) with red open circles and the LiLACS NZ cohort (257 men from a distinct geographical area to Otago) with green open circles. Each circle represents an individual. A: The Vascular cohort had a higher mean AMH value than the aged matched portion of Otago cohort (p<0.001), but were not different to the younger subset (20–50 years), p = 0.35. B: The vascular cohort had similar InhB levels to the aged matched subset of the Otago cohort (p = 0.13), but lower than levels than the younger Otago subset (p<0.001). C: The LiLACS NZ cohort showed similar aged related decline in both A: AMH (p = 0.08) and D: InhB (p = 0.76) when compared to the aged matched subset of the Otago cohort, whilst being different to the younger subset for both hormones, p<0.001 (20–50 years).

The Otago cohort was then compared to two other cohorts of elderly men, to determine the generalizability of the observations. The first comparison was between the community-dwelling Dunedin men, described above, and a second Dunedin cohort (n = 153) that had been selected for the absence of cardiovascular disease (vascular cohort). The levels of AMH in the vascular cohort were higher than the age-matched portion of the Otago cohort (Fig. 4A, p<0.001) and were not different from the younger part of the Otago cohort (Fig. p = 0.35). In contrast, the Otago and the vascular cohorts had similar age-related decline in InhB values.

The elderly portion of the Otago cohort (n = 99) was then compared with the LiLACS NZ cohort (n = 257), which was collected from a more northern community, with a different socioeconomic and ethnic composition. The age-matched AMH and InhB levels were not different between the cohorts, although 3.5% of the LiLACS NZ cohort had atypically high levels of InhB, which could not be attributed to race (Fig. 4D).

**Figure 5:**
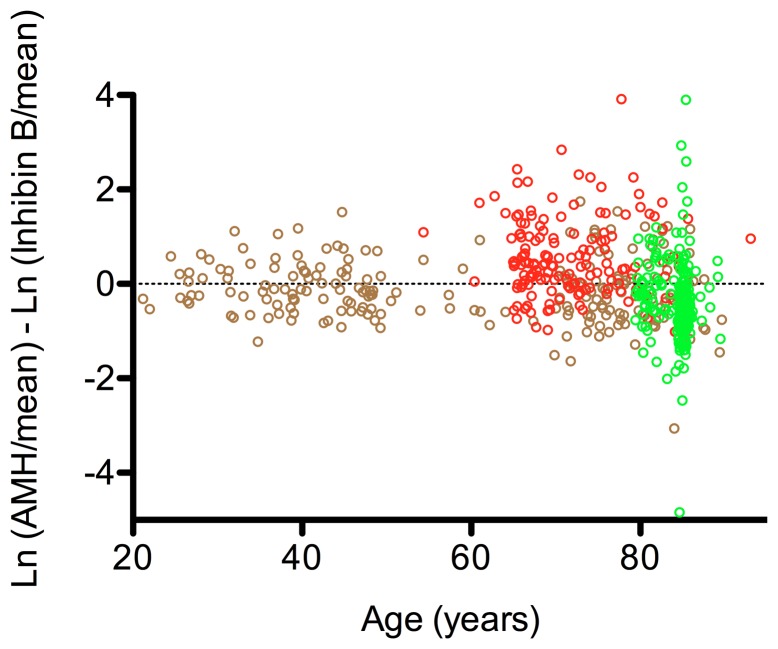
The ratio of AMH and InhB in the cohorts of community dwelling men. The Otago cohort is represented by brown open circles (n = 205), the vascular cohort by red open circles (n = 153) and the LiLACS NZ cohort by green open circles (n = 257). Each open circle represents an individual. An increasing divergence was seen in the older age groups. The vascular cohort had an AMH bias. The ratio of the hormones across the age spectrum was calculated as described in the Methods.

When the three cohorts were combined, the elderly men displayed larger divergence in the ratio of AMH and InhB, in both the positive and negative directions. The vascular group had a prominent AMH bias (Fig. 5).

## Discussion

This study shows that the hormonal output of Sertoli cells persists in the very old, but with characteristics that differ on average from younger men. The levels of both AMH and InhB were highly variable in the elderly men, with mean values approximating half those of younger men. This is consistent with histological studies of the testes showing age-related attrition of Sertoli cells [20], [21]. A small minority of men had minimum levels of both AMH and InhB, indicating a near complete cessation of their Sertoli cell output. In this respect, these men are similar to post-menopausal women, who lose AMH and InhB due to regression of their ovarian follicular cells [26], [27], [28].

There was an age-related increase in the inter-person variation in the levels of AMH and InhB, suggesting that the regulation of Sertoli cell output changes in some elderly men. A dysynchrony between LH and testosterone occurs in elderly men [29]. Consequently, alteration of the gonadal-pituitary axis may also be important for age-related changes in Sertoli cell hormones, as FSH regulates InhB [30] and as AMH is putatively under the control of GnRH [31]. LH and FSH exhibit a circhoral rhythm in men [32], whereas AMH has a half-life of greater than a day [33]. Hence, repeated hormonal measurements of individual will be needed to examine this hypothesis, rather than the cross-sectional approach used in this study.

The vascular cohort, which was free from cardiovascular disease, had higher levels of AMH and tended to have a higher AMH to InhB ratio. The most obvious difference between the cohorts is the proven absence of cardiovascular disease in the vascular cohort. The importance of this is currently unknown, although it is consistent with recently observations linking AMH to atherosclerosis in rhesus monkeys [34] and to the diameter of the abdominal aorta in health men [12].

As noted previously, the levels of InhB were lower in older men, with high statistical significance. This contrasts with a previous study by Mahmoud et al, who reported higher InhB levels in the elderly, with minimal age-related deterioration [22]. Mahmoud et al’s study excluded men being treated for diabetes mellitus, whereas the current study includes a more representative cross-section of elderly men. Low AMH in younger adults has also been associated with metabolic syndrome [35]. If Sertoli cell function is causally linked to insulin resistance, then changes in Sertoli cell output may contribute to age-related increase in the incidence of Type 2 diabetes mellitus and related complications. This points to the need for epidemiological studies examining the relationship between AMH levels and health status.

Secular changes have been observed in the androgen status of elderly men, with men born in earlier generations having higher levels of testosterone than more recent generations [36]. The current study is cross-sectional, and we therefore do not exclude the possibility that some of the observed age-related changes in Sertoli hormones also arise from intergenerational differences.

In summary, this study suggests that the plasma levels of Sertoli hormones decline with aging, but with high variability. There are associations between AMH, InhB and the ratio of AMH/InhB to the health status of elderly men. This identifies an area for future research, particularly into the role of Sertoli hormones in cardiovascular disease and frailty in elderly men.

## Supporting Information

Figure S1 Distribution of AMH levels in men. A: the young subset of community dwelling men in Otago (n = 106, 19–67 years), B: the older subset in Otago (n = 99, 70–90 years). Both distributions were positively skewed (skewness 1.35 for young subset (p<0.001), 2.06 for older subset (p<0.001)). (TIF)

Figure S2 Distribution of InhB in men. A: young subset of community dwelling men in Otago (n = 106, 19–67 years), B: older subset in Otago (n = 99, 70–90 years). The young subset cohort was normally distributed, but the older cohort was positively skewed (skewness 0.04 for young subset (p = 0.85), 0.11 for older subset (p = 0.64)). (TIF)
