# Correction: Elderly Men Have Low Levels of Anti-Müllerian Hormone and Inhibin B, but with High Interpersonal Variation: A Cross-Sectional Study of the Sertoli Cell Hormones in 615 Community-Dwelling Men

**DOI:** 10.1371/annotation/d6097df2-37ed-4561-8885-f54dcaa3aeb6

**Published:** 2013-10-11

**Authors:** Yih Harng Chong, Nicola A. Dennis, Martin J. Connolly, Ruth Teh, Gregory T. Jones, Andre M. van Rij, Stephanie Farrand, A. John Campbell, Ian S. McLennan

There was an error in the name of the ninth author.

The correct name of this author is: Ian McLennan 

