# Supplementary figures and images for: Elderly Men Have Low Levels of Anti-Müllerian Hormone and Inhibin B, but with High Interpersonal Variation: A Cross-Sectional Study of the Sertoli Cell Hormones in 615 Community-Dwelling Men
Source: PLoS One. 2013 Aug 5;8(8):e70967. doi: 10.1371/journal.pone.0070967 (PMC3733803; doi:10.1371/journal.pone.0070967)

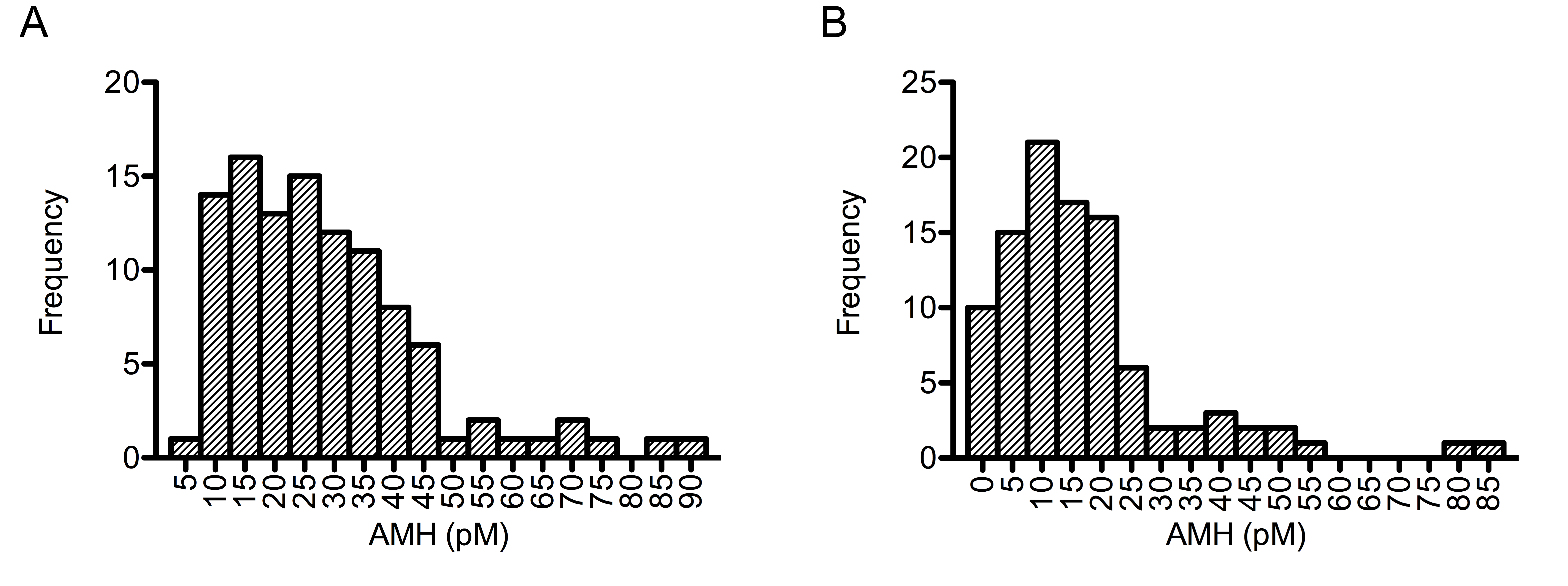

Supplement: Figure S1 — (TIF) [file pone.0070967.s001.tif]

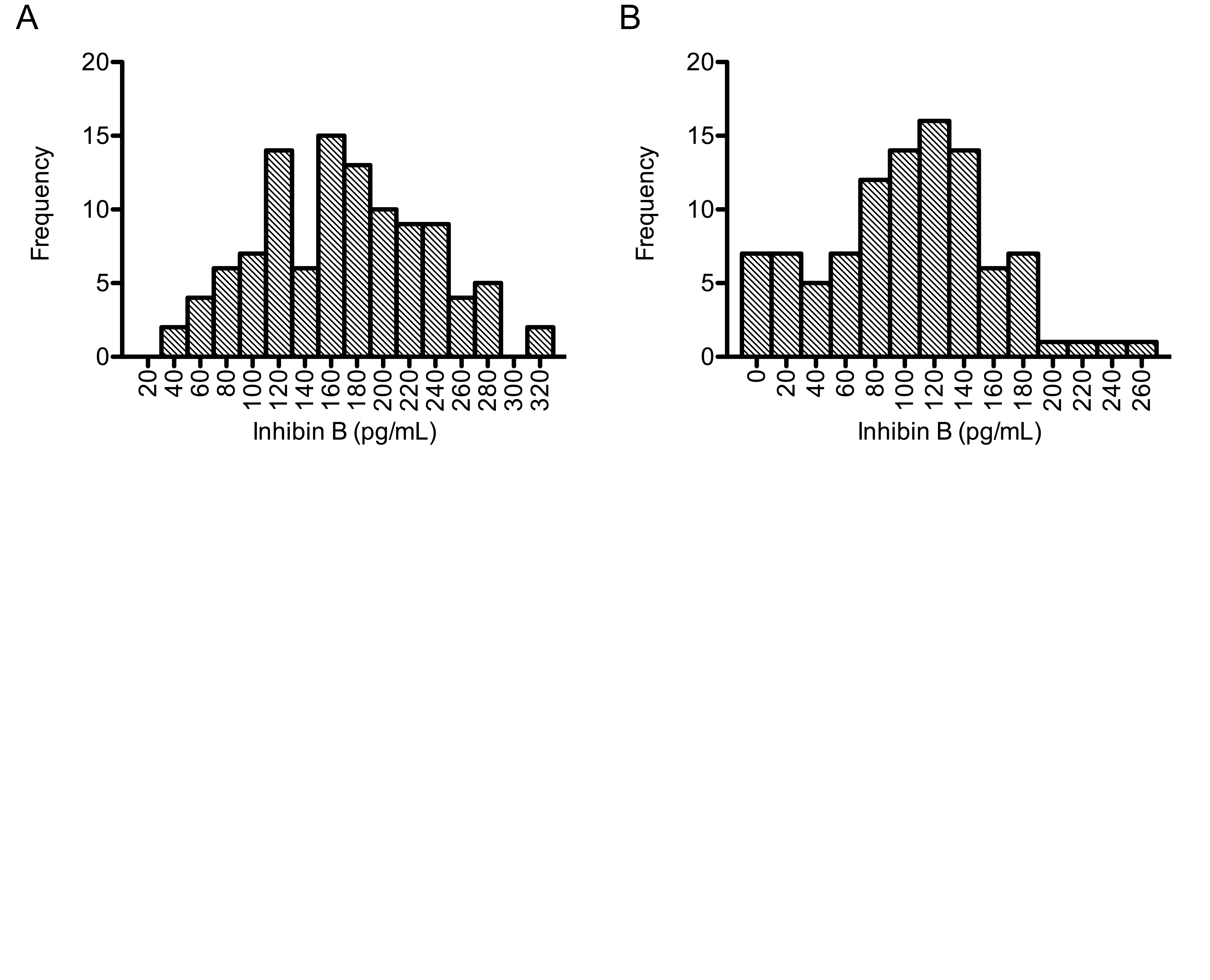

Supplement: Figure S2 — (TIF) [file pone.0070967.s002.tif]
